# Examinations for Life Insurance

**Published:** 1867-07

**Authors:** 


### Editorial Department.

### Examinations for Life Insurance.

The habit of Life Insurance has grown so greatly in popular favor, that at the present time the great majority of the business portion of large cities invest in it a portion of their yearly incomes. Life Insurance Companies are offering inducements which attract even the wealthier men who obtain insurance upon their lives as an investment. This popular belief in its safety and propriety, has given organization to a great many companies, and large amounts of capital stock are now invested in the business of life insurance.

It is not our purpose to speak of its advantages, or go into any review of the general questions involved in it; every one is sufficiently familiar with its protective advantages to the poor, and the rich would not be likely to invest in it, if any objections were really well founded. It is, however, only upon the physician's certificate, and its importance to the companies making insurance, that we propose any remarks, and our suggestions will be directed to both physicians and insurance companies.

Insurance Companies, base a contract upon the probability of life, in the assured; they propose to take his chances of living to the age of sixty-five, more or less, and if he fails of this standard they lose, if life is prolonged beyond it, they gain. Physicians are appointed to examine into the physical condition, and are expected by companies to do this, with great fidelity aud care. It is obvious that everything depends upon the discrimination, intelligence and faithfulness with which this examination is made, and that companies who allow careless or incompetent examinations by their examining physicians, are not wise for themselves or safe for the community. We have a personal prejudice in favor of insurance, and believe it a duty every one, not above its necessity, owes to those dependent upon them for support; but our confidence in its safety and long continuance has been greatly weakened by observing how insufficient and unsatisfactory are the examinations upon which it is based. We have known consumptives recommended as first rate risks, in the later stages of the disease, and we have known cardiac diseases, certain to terminate fatally in a few years, entirely overlooked, to say nothing of the numerous instances of insurance upon the lives of confirmed inebriates, sometimes delirious even, at the time of examination. That these are exceptional, rather than common cases, is certainly to be hoped; they are sufficiently frequent to throw suspicion upon the permanency of insurance companies, and must already have resulted in serious loss, and greatly augmented the amount of premium which would be required if first class risks, only, were taken. Why is this, and what interests have physicians in it?

The usual compensation for physical examination sufficient to base a rational therapeutical conclusion upon, is from five to ten dollars. The usual price paid by insurance companies for an opinion upon which they mainly base their risks, is from two to five dollars, perhaps three is about the average. Companies show a stupidity of management in this respect truly astonishing, and physicians show a corresponding insensibility as to what rightfully belongs to them, and no wonder that the "blind leaders of the blind, both fall into the ditch." The manner of appointing physicians to insurance companies, where really so much is involved, shows a lack of common discrimination, and we are daily reminded of its absurdity; it runs thus: Dr. —— having applied for appointment as examining physician to ——— Life Insurance Company, and referred to you as to 'ability and capacity, we desire to know your opinion of his fitness for such office, will he serve us satisfactorily? Yes, being the shortest and safest word, we invariably write it after the question, and return the document. We have no reason for writing anything else, and should not feel at liberty to use any other word unless we were prepared to sustain the opinion by adequate testimony. *All* who receive such appointments obtain *such* testimonials of character and standing. When the question is, who in your city or county will give us the safest and best opinion? who do you suggest as most capable of doing us this serviee? the whole subject becomes changed, and insurance companies show they are not gambling with other people's money.

This whole thing may be expressed in few words. Capable physicians, whose opinions are worth anything, should never give them where so much depends upon their decisions, and where there is so great ability to pay, without adequate compensation. It is extremely illiberal as well as shortsighted for insurance companies to offer for this service insufficient compensation, and it is allowing low estimate to be placed upon important service, for physicians to accept it, if it is offered. We advise the profession to make their examinations worth to the companies a respectable compensation, and to accept nothing else. We would also suggest to companies the danger of accepting risks upon the recommendation of any physician who values his time and opinions cheaply. One other sabject connected with life insurance and we have done our duty to both parties.

Insurance Companies are in the habit of asking family physicians to certify to certain facts, without any compensation at all. The knowledge a family physician may possess is often of great value in determining the safety of a risk, and they can never obtain it without adequate compensation. Physicians have no inducement to communicate their knowledge of their patients to insurance companies, and the non-committal manner in which the questions are for the most part answered, show, or should show companies, that as the matter now stands, it is the purpose *not* to know anything about it. If our patients have had constitutional diseases, or bad habits, it is greatly for the interest of the family physician to keep his knowledge to himself, and insurance companies are rarely made wiser by his answers to their usual questions. It will be replied, that this statement is given in the interest of the patient, and that the value of it should be paid by the assured. This is not true; it is the *company* who desire the knowledge an attending physician may possess, and they should be expected to pay fairly, to obtain it. As the custom now is, with some companies, they pay a mere pittance for exam ination by their own physician, depend considerably upon family physician's replies, and really obtain nothing from either, worth a farthing in correctly estimating the physical soundness, or the probable duration of life.

